# One- and two-year structural changes of mavacamten therapy in hypertrophic obstructive cardiomyopathy: a case report with serial comprehensive CMR demonstrating continuous reverse remodelling

**DOI:** 10.1093/ehjcr/ytag165

**Published:** 2026-03-09

**Authors:** Katharina Seuthe, Roman Pfister, Carl-Hubertus Schönherr, Kenan Kaya, Lenhard Pennig

**Affiliations:** Department III of Internal Medicine, Heart Center, Faculty of Medicine and University Hospital Cologne, University of Cologne, Kerpener Straße 62, Cologne 50937, Germany; Department III of Internal Medicine, Heart Center, Faculty of Medicine and University Hospital Cologne, University of Cologne, Kerpener Straße 62, Cologne 50937, Germany; Practice Dr. Schönherr—General Medicine, Allergology, and Chiropractic Therapy in the South of Cologne, Rodenkirchener Str. 158, Cologne 50997, Germany; Institute for Diagnostic and Interventional Radiology, Faculty of Medicine and University Hospital Cologne, University of Cologne, Kerpener Straße 62, Cologne 50937, Germany; Institute for Diagnostic and Interventional Radiology, Faculty of Medicine and University Hospital Cologne, University of Cologne, Kerpener Straße 62, Cologne 50937, Germany

**Keywords:** Hypertrophic obstructive Cardiomyopathy, Myosin-Inhibitor, Cardiac magnetic resonance, Case report

## Abstract

**Background:**

Hypertrophic obstructive cardiomyopathy (HOCM) is characterized by dynamic left ventricular outflow tract (LVOT) obstruction and impaired quality of life. Mavacamten, a first-in-class myosin inhibitor, offers a novel therapeutic approach for HOCM, which improves clinical symptoms and exercise capacity while leading to reduction of LVOT gradient and favourable cardiac remodelling in echocardiography and cardiovascular magnetic resonance (CMR). However, data on CMR-derived treatment effects remain limited to short follow-up studies.

**Case summary:**

A 39-year-old male with symptomatic HOCM refractory to bisoprolol and disopyramide was initiated on mavacamten therapy. Serial CMR after one and two years demonstrated progressive reverse remodelling over the follow-up period, including ongoing reduction of LV ejection fraction and LV mass index. While late gadolinium enhancement mass remained unchanged, there were divergent changes to measures of interstitial fibrosis: While native T1 relaxation times steadily decreased, extracellular volume (ECV) fraction initially increased but normalized at two-year follow-up.

**Discussion:**

This case indicates that mavacamten leads to longer-term cardiac remodelling up to two years after initiation of treatment, beyond early haemodynamic improvement and reduction of LVOT obstruction. These observations nurture additional studies to investigate longer-term effects on myocardial structure, function, and interstitial fibrosis.

Learning points

- Mavacamten induces sustained reverse myocardial remodelling beyond one year in obstructive hypertrophic cardiomyopathy, as shown by serial cardiovascular magnetic resonance.
- Cardiovascular magnetic resonance is essential for diagnosis and treatment monitoring in obstructive hypertrophic cardiomyopathy.

## Introduction

Hypertrophic obstructive cardiomyopathy (HOCM) is a genetic disorder, most commonly caused by variants in sarcomere protein genes, and characterized by myocardial hypertrophy with dynamic left ventricular outflow tract (LVOT) obstruction. Conventional negative inotropic therapy—including beta-blockers, calcium channel blockers (CCBs), and disopyramide—remains the first-line treatment, yet many patients continue to experience persistent symptoms and disease progression.^1^

The advent of mavacamten, a selective cardiac myosin inhibitor, has introduced a targeted approach to reduce hypercontractility and LVOT obstruction, subsequently improving New York Heart Association (NYHA) class and exercise capacity.^2^ According to current guidelines, mavacamten is now recommended (class IIa) for symptomatic patients with HOCM and an LVOT gradient ≥50 mmHg despite optimal baseline therapy.^1^ While longer-term functional and clinical benefits of mavacamten up to three years after treatment initiation are well documented,^3^ studies investigating reverse remodelling of myocardial structure using transthoracic echocardiography (TTE)^4,5^ and cardiovascular magnetic resonance (CMR) are limited to short-term follow-up up to one year.^6,7^

This case illustrates the sustained efficacy of mavacamten, highlighting its ongoing effect of reverse remodelling and fibrosis regression as documented by serial CMR imaging.

## Summary figure

**Figure:**
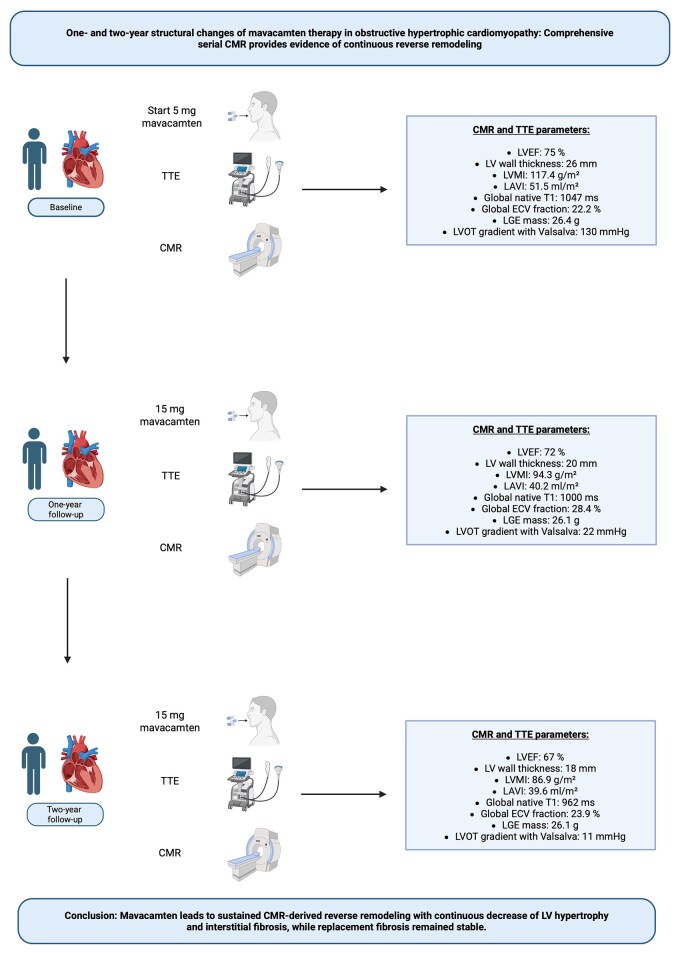
CMR, cardiovascular magnetic resonance; ECV, extracellular volume; LGE, late gadolinium enhancement; LV, left ventricular; LAVI, left atrial volume index; LVEF, left ventricular ejection fraction; LVMI, left ventricular mass index; LVOT, left ventricular outflow tract; TTE, transthoracic echocardiography Created in BioRender. Kaya, K. (2025) https://BioRender.com/oro25a7

## Case presentation

A 39-year-old male diagnosed with HOCM five years earlier presented to our tertiary referral centre with progressive dyspnoea (NYHA class III–IV) and dizziness, despite treatment with bisoprolol (10 mg/day) and disopyramide (600 mg/day). On physical examination, a systolic murmur was audible along the left sternal border. Vital signs were stable (blood pressure 126/80 mmHg, heart rate 68 bpm), but the patient yielded a high body mass index (BMI) of 33 kg/m^2^. NT-proBNP and troponin T levels were elevated at 950 pg/mL and 0.053 µg/L, respectively. There was no history of syncope, atrial fibrillation, or sudden cardiac death (SCD) in the family. Ambulatory Holter monitoring revealed no non-sustained ventricular tachycardia. According to the ESC HCM Risk-SCD model, the estimated 5-year risk of SCD was low (3.7%).

**Figure 1:**
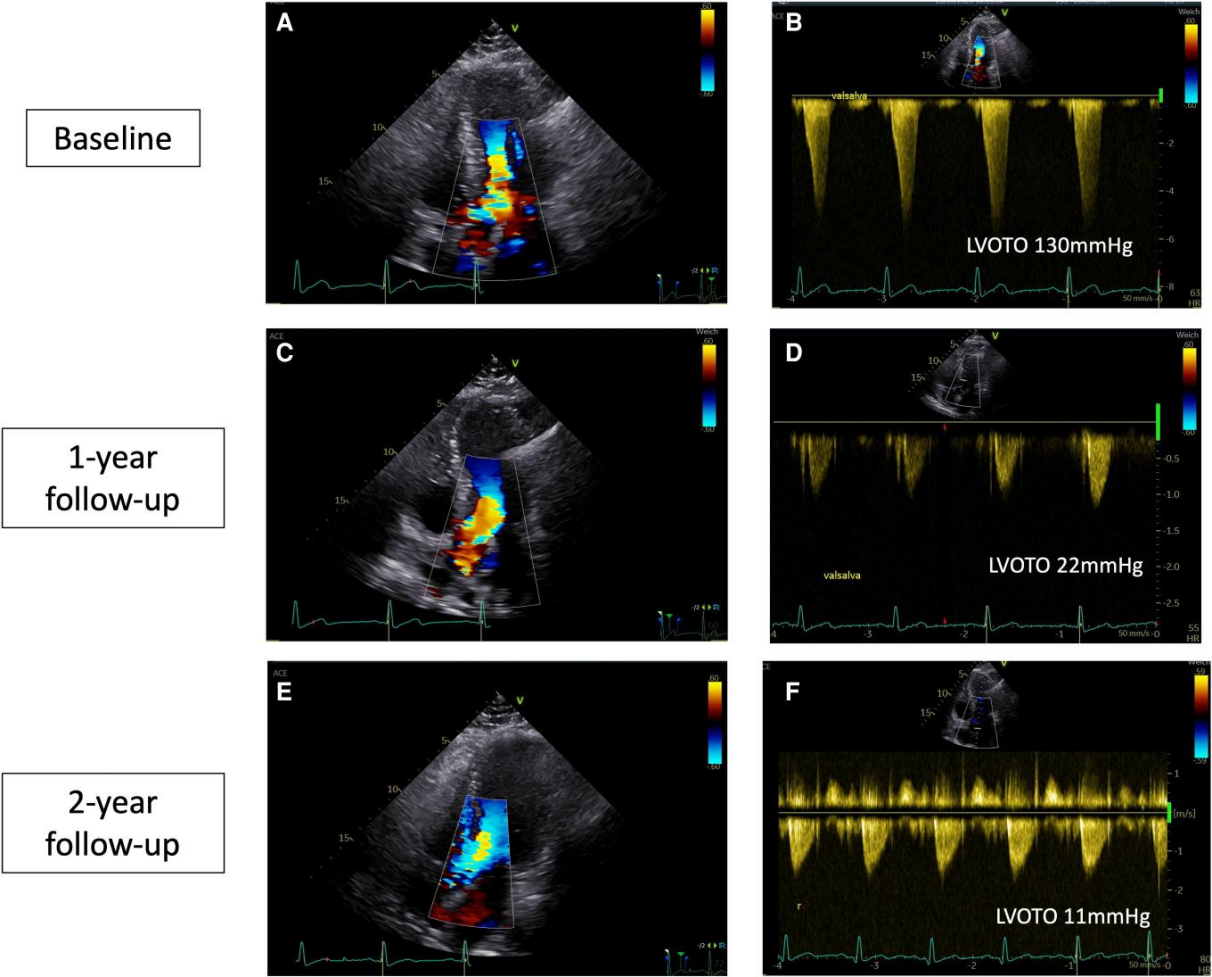
Functional effect on Valsalva left ventricular outflow graft obstruction under mavacamten shown in serial transthoracic echocardiography examinations. Colour Doppler in four-chamber view (A, C, and E) and continuous wave Doppler images (B, D, and F) are depicted. LVOTO = left ventricular outflow graft obstruction.

**Figure 2:**
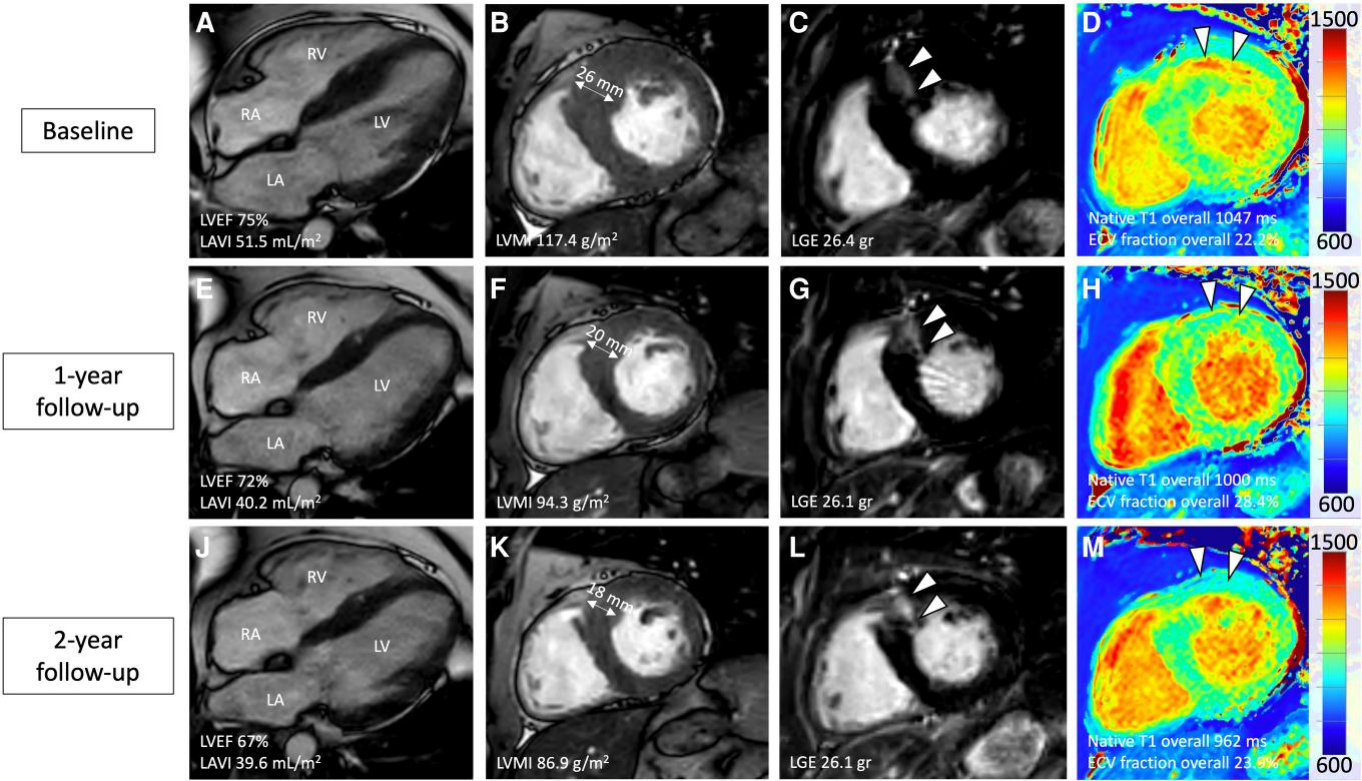
Effects of mavacamten treatment as shown in serial cardiovascular magnetic resonance examinations at 1.5 T in a 42-year-old man with hypertrophic obstructive cardiomyopathy (A–M). Four-chamber (A, E, and J) and short-axis (B, F, and K) cine, short-axis late gadolinium enhancement (LGE; C, G and L), and short-axis native T1 mapping (D, H, and M) sequences are depicted. Note the continuous decrease of maximal left ventricular (LV) wall thickness, LV mass index (LVMI), and LV ejection fraction (LVEF) over time. While left atrial volume index (LAVI) decreased from baseline to one-year follow-up, there were no changes between follow-ups. Replacement fibrosis as depicted in LGE was stable over time (C, G, and L; arrowheads). However, there was a strong reduction of interstitial fibrosis as measured by native T1 from baseline to one- and two-year follow-ups, the latter yielding values within institute reference ranges (961–985 ms). Note the strong reduction of T1 values at the basal anterior and anteroseptal segments over time (D, H, and M; arrowheads). While ECV fraction increased at 1-year follow-up to the upper limit of reference values at 1.5 T (17–29%), values decreased and normalized at 2-year follow-up. RA = right atrium, RV = right ventricle.

TTE revealed asymmetric septal hypertrophy and a LVOT gradient with Valsalva of 130 mmHg (*Figure 1*) with systolic anterior motion (SAM) of the mitral valve and moderate mitral regurgitation. Electrocardiogram demonstrated sinus rhythm with criteria for left ventricular hypertrophy. CMR was performed using a 1.5 T system (*Figure 2*) and confirmed LV hypertrophy with markedly increased indexed LV mass (117.4 g/m^2^) and maximal wall thickness (26 mm) while left ventricular ejection fraction (LVEF) was within higher normal limits (75%). Late gadolinium enhancement (LGE) revealed replacement fibrosis in the basal and midventricular septum (mass of 26.4 g). Global native T1 values were elevated (1047 ms), indicating diffuse interstitial fibrosis, while the extracellular volume (ECV) fraction was within lower normal limits at 22.2%.^8^

Given persistent symptoms and evidence of disease progression despite optimal medical therapy, treatment with mavacamten was initiated in accordance with ESC 2023 guidelines. Pharmacogenetic testing identified a rapid metabolizer phenotype, and therapy was started at 5 mg/day following down-titration and discontinuation of disopyramide, respectively. The mavacamten dose was gradually increased to 10 and 15 mg/day in 12-week intervals, with TTE-monitoring every 4 weeks.

The patient reported early symptomatic improvement: after 12 weeks of treatment, the NYHA class improved to II, and the symptoms of dizziness had resolved. Regarding haemodynamic response, the LVOT Valsalva gradient decreased to <30 mmHg after 8 weeks on the maximum available dose of 15 mg mavacamten. No adverse effects were noted during the course of therapy.

At one-year follow-up, TTE demonstrated resolution of SAM and mitral regurgitation with a Valsalva LVOT gradient of 22 mmHg (*Figure 2*). Moreover, CMR revealed substantial structural reverse remodelling: maximal septal thickness decreased to 20 mm and indexed LV mass declined to 94.3 g/m^2^. LVEF only slightly decreased from 75% to 72%, whereas left atrial volume index (LAVI) drastically decreased from 51.5 mL/m^2^ to 40.2 mL/m^2^. However, changes to measures of interstitial fibrosis were divergent: Whereas global native T1 values decreased to 1000 ms, ECV fraction increased to 28.4% (*Figure 2*). However, LGE burden remained unchanged at 26.1 g.

After two years of continuous mavacamten therapy (15 mg/day), the patient remained clinically stable (NYHA class II), with sustained relief of LVOT obstruction (11 mmHg). However, CMR demonstrated further reverse remodelling compared to the one-year follow up (*Figure 2*): maximal wall thickness and LV mass index further decreased to 18 mm and 86.9 g/m^2^, respectively, while LVEF now yielded normal values (67%), whereas LAVI remained widely unchanged (39.6 mL/m^2^). Notably, native T1 values showed a further reduction (962 ms) towards lower reference ranges at 1.5 T^8^ while ECV fraction decreased with values comparable to baseline CMR (23.9%). LGE burden remained unchanged at 26.1 g. No adverse cardiac events were recorded during the 2-year follow-up.

## Discussion

This case illustrates the long-term reverse remodelling of mavacamten in HOCM with notable effects on myocardial structure, function, and fibrosis between one- and two-year follow-up. Beyond its well-established effect on LVOT gradient reduction and relief of clinical symptoms,^9^ mavacamten therapy was associated with sustained and ongoing reverse remodelling in CMR—including ongoing reductions of maximal LV wall thickness, LV mass index, and measures of interstitial fibrosis as assessed by native T1 mapping.

To our knowledge, this is the first case report to describe serial CMR findings over a two-year course of myosin inhibition therapy. While previous studies have shown promising structural changes after 30 weeks^6^ and one year^7^ it remains unclear whether such remodelling progresses or plateaus over time. In this case, we observed a sustained reduction in LV mass index and wall thickness, established determinants of outcome in HCM,^10,11^ as they predict adverse events such as ventricular arrhythmias, sudden cardiac death, and new-onset atrial fibrillation. Notably, continued remodelling beyond one year was evident, culminating in normalization of native T1 values,^8^ which suggests the potential reversal of diffuse interstitial fibrosis—an independent risk factor in HCM.^12^

LGE depicts replacement fibrosis in HCM, the presence and extent of which are strongly associated with disease progression and the risk of sudden cardiac death,^13^ making it a component of risk stratification. As expected, and in line with current literature,^6,7^ replacement fibrosis remained stable over time; reinforcing the notion that focal fibrosis is largely unmodifiable, even over prolonged follow-up. This stability on the other hand, may underscore the potential benefits of early treatment, especially given that no further increase in LGE was observed—contrary to the reported average progression rate of fibrosis in HCM (+0.5% annually).^14^ ECV fraction, which represents the extracellular components (e.g. extracellular matrix and interstitium),^8^ is another parameter of tissue characterization with strong prognostic impact in HCM.^15^ Mavacamten treatment initially led to increased ECV fraction after one year, which might seem counter-intuitive given the decrease of native T1 indicating extracellular fibrosis. These findings can be explained by the strong reduction of LV mass corresponding to the myocellular compartment leading to an expansion of the ECV. However, these effects normalized at second year follow-up.

Although this single-patient experience is encouraging, larger longitudinal studies are needed to confirm these findings and better understand the trajectory of remodelling under long-term mavacamten therapy in addition to symptomatic relief. Treatment is currently limited to patients with HOCM and resting or provoked LVOT gradients ≥50 mmHg, who remain symptomatic despite optimal medical therapy.^1^ Moreover, our case highlights the important role of CMR in diagnosis and therapy surveillance in HCM, given its visualization of changes to myocardial structure and tissue, which are not detectable by echocardiography, as recommended by current ESC guidelines for cardiomyopathies (class I).^1^

## Patient perspective

The patient reported significant improvement in symptoms, functional capacity, and overall quality of life under the treatment. He was able to resume playing football with his two sons (age 6 and 8), which he considered a major personal milestone. He expressed satisfaction with the non-invasive nature of the treatment and valued the close imaging-based follow-up, which provided reassurance about the therapy's effectiveness.

## Data Availability

The data from this study are available from the corresponding author upon reasonable request.

## Lead author biography

Katharina Seuthe, M.D., is a consultant cardiologist at the University Hospital of Cologne, Germany. She specialized clinically and in research in heart failure, cardiomyopathies, and cardiovascular imaging.

## Notes

**Consent:** Written informed consent was obtained from the patient for publication of this case

## References

[1] Arbelo E, Protonotarios A, Gimeno JR, Arbustini E, Barriales-Villa R, Basso C, et al 2023 ESC guidelines for the management of cardiomyopathies. Eur Heart J 2023;44:3503–3626. 37622657 10.1093/eurheartj/ehad194

[2] Olivotto I, Oreziak A, Barriales-Villa R, Abraham TP, Masri A, Garcia-Pavia P, et al Mavacamten for treatment of symptomatic obstructive hypertrophic cardiomyopathy (EXPLORER-HCM): a randomised, double-blind, placebo-controlled, phase 3 trial. The Lancet 2020;396:759–769. 10.1016/S0140-6736(20)31792-X 32871100

[3] Garcia-Pavia P, Oręziak A, Masri A, Barriales-Villa R, Abraham TP, Owens AT, et al Long-term effect of mavacamten in obstructive hypertrophic cardiomyopathy. Eur Heart J 2024;45:5071–5083. 39217450 10.1093/eurheartj/ehae579 PMC11646600

[4] Desai MY, Owens A, Wolski K, Geske JB, Saberi S, Wang A, et al Mavacamten in patients with hypertrophic cardiomyopathy referred for septal reduction: week 56 results from the VALOR-HCM randomized clinical trial. JAMA Cardiol 2023;8:968–977. 37639243 10.1001/jamacardio.2023.3342 PMC10463171

[5] Desai MY, Okushi Y, Gaballa A, Wang Q, Geske JB, Owens AT, et al Serial changes in ventricular strain in symptomatic obstructive hypertrophic cardiomyopathy treated with mavacamten: insights from the VALOR-HCM trial. Circ Cardiovasc Imaging 2024;17:e017185. 39221824 10.1161/CIRCIMAGING.124.017185 PMC11410149

[6] Saberi S, Cardim N, Yamani M, Schulz-Menger J, Li W, Florea V, et al Mavacamten favorably impacts cardiac structure in obstructive hypertrophic cardiomyopathy: EXPLORER-HCM cardiac magnetic resonance substudy analysis. Circulation 2021;143:606–608. 33190524 10.1161/CIRCULATIONAHA.120.052359

[7] Seuthe K, Pfister R, Gertz RJ, Ten Freyhaus H, Janssen JP, Kural M, et al One-year functional and structural effects of mavacamten in obstructive hypertrophic cardiomyopathy: a comprehensive CMR study. Eur Heart J Cardiovasc Imaging 2025;26:1685–1687. 40796166 10.1093/ehjci/jeaf231

[8] Kawel-Boehm N, Hetzel SJ, Ambale-Venkatesh B, Captur G, Francois CJ, Jerosch-Herold M, et al Reference ranges (“normal values”) for cardiovascular magnetic resonance (CMR) in adults and children: 2020 update. J Cardiovasc Magn Reson 2020;22:1–63. 33308262 10.1186/s12968-020-00683-3 PMC7734766

[9] Rader F, Oręziak A, Choudhury L, Saberi S, Fermin D, Wheeler MT, et al Mavacamten treatment for symptomatic obstructive hypertrophic cardiomyopathy: interim results from the MAVA-LTE study, EXPLORER-LTE cohort. JACC Heart Fail 2024;12:164–177. 38176782 10.1016/j.jchf.2023.09.028

[10] Olivotto I, Maron MS, Autore C, Lesser JR, Rega L, Casolo G, et al Assessment and significance of left ventricular mass by cardiovascular magnetic resonance in hypertrophic cardiomyopathy. J Am Coll Cardiol 2008;52:559–566. 18687251 10.1016/j.jacc.2008.04.047

[11] Dohy Z, Szabo L, Toth A, Czimbalmos C, Horvath R, Horvath V, et al Prognostic significance of cardiac magnetic resonance-based markers in patients with hypertrophic cardiomyopathy. Int J Cardiovasc Imaging 2021;37:2027–2036. 33555536 10.1007/s10554-021-02165-8 PMC8255255

[12] Wang J, Zhang J, Liu W, Pu L, Qi W, Xu Y, et al Prognostic value of myocardial T1 mapping for predicting adverse events in hypertrophic cardiomyopathy. Circ Cardiovasc Imaging 2025;18:e017174. 39957669 10.1161/CIRCIMAGING.124.017174

[13] Kiaos A, Daskalopoulos GN, Kamperidis V, Ziakas A, Efthimiadis G, Karamitsos TD. Quantitative late gadolinium enhancement cardiac magnetic resonance and sudden death in hypertrophic cardiomyopathy. JACC Cardiovasc Imaging 2024;17:489–497. 37632503 10.1016/j.jcmg.2023.07.005

[14] Raman B, Ariga R, Spartera M, Sivalokanathan S, Chan K, Dass S, et al Progression of myocardial fibrosis in hypertrophic cardiomyopathy: mechanisms and clinical implications. Eur Heart J Cardiovasc Imaging 2019;20:157–167. 30358845 10.1093/ehjci/jey135 PMC6343081

[15] Li Y, Liu X, Yang F, Wang J, Xu Y, Fang T, et al Prognostic value of myocardial extracellular volume fraction evaluation based on cardiac magnetic resonance T1 mapping with T1 long and short in hypertrophic cardiomyopathy. Eur Radiol 2021;31:4557–4567. 33449190 10.1007/s00330-020-07650-7

